# Primary Care Peer-Supported Internet-Mediated Psychological Treatment for Adults With Anxiety Disorders: Mixed Methods Study

**DOI:** 10.2196/19226

**Published:** 2020-08-20

**Authors:** Linnea Nissling, Claudia Fahlke, Josefine L Lilja, Ingmarie Skoglund, Sandra Weineland

**Affiliations:** 1 Department of Psychology University of Gothenburg Gothenburg Sweden; 2 R&D Primary Health Care Västra Götaland Sweden; 3 General Medicine, School of Public Health and Community Medicine, Institute of Medicine Sahlgrenska Academy University of Gothenburg Gothenburg Sweden

**Keywords:** iCBT, cognitive behavioral therapy, internet-based intervention, anxiety disorders, primary care, treatment adherence and compliance, peer support

## Abstract

**Background:**

The effect of internet-delivered cognitive behavioral therapy (iCBT) on anxiety in adults is well-known. However, patient dropouts and poor adherence to treatment are common. Feelings of belonging and empowerment from the treatment might be key to the completion of iCBT. Peer support workers are people with a personal experience of mental health problems, trained to provide professional support to people who require mental health care.

**Objective:**

This study aims to assess patient experiences; the feasibility, safety, and acceptability; and preliminary effectiveness on anxiety and depression, empowerment, and adherence to treatment in an 8-week peer-supported iCBT program for patients with anxiety disorders treated in primary care.

**Methods:**

This was a single-arm mixed methods feasibility study. Participants were patients referred to a central unit for iCBT in primary care. Quantitative data were collected pre-, post-, and 3 months postintervention. Qualitative data were collected through semistructured interviews.

**Results:**

A total of 9 participants completed the quantitative outcome assessment. Statistically significant improvements were observed in perceived empowerment at a 3-month follow-up, and significant decreases in anxiety, depression, and psychological distress at the end of the treatment were maintained at a 3-month follow-up. In total, 8 of the 9 patients showed improvement in the severity of their symptoms of anxiety. Adherence to treatment was good among the participants. No serious adverse events were reported. Eight participants were enrolled in the qualitative analysis. The qualitative results showed 3 main themes: (1) real contact in an online world, (2) empowering experiences, and (3) being behind the wheel. Qualitative results largely emphasized the personal relationship and supported the acceptability of adding peer support to iCBT.

**Conclusions:**

Peer support in digital treatment seems to be a safe and acceptable intervention. The preliminary results suggest the effectiveness of peer support on patient empowerment, anxiety, depression, psychological distress, and adherence to treatment. The results indicate the need for future studies to evaluate the effect of adding peer support to iCBT in larger randomized controlled trials.

## Introduction

### Background

Anxiety disorders are mental health problems commonly encountered in primary care that may lead to impaired functioning in daily life. Data from the Swedish National Public Health Survey show that in 2018, 39% of adult respondents reported symptoms of anxiety [1]. Anxiety disorders are mainly treated with antidepressants in combination with various psychological treatments; however, the lack of access to such treatments is a general problem in Sweden’s routine health care [2].

Cognitive behavioral therapy (CBT) is a well-documented and effective method for various anxiety disorders [3] and is often recommended as a first-hand treatment in Sweden [2]. Therapist-guided internet-delivered cognitive behavioral therapy (iCBT) may be an effective medium for disseminating psychological treatment by providing greater availability and reaching out to patients who would otherwise not seek care due to fear of stigmatization [4]. Studies show that iCBT is effective for treating a number of psychiatric disorders, including several anxiety disorders, depression, substance abuse, and bipolar disorder, among others [5-8]. A growing number of studies also show that the effects of such treatment persist at long-term follow-ups [8-11] and that it may be as efficient as face-to-face therapy [7,12]. Moreover, people who have completed iCBT generally show a positive attitude toward the treatment and its effectiveness [13]. However, more research is needed on the efficacy of iCBT in clinical settings, as only a limited number of studies have been conducted to date [12].

Despite the positive results shown for iCBT, patient dropouts and poor adherence to treatment are common [14-16]. To increase patient compliance and completion of iCBT, additional interventions might be needed to help patients cope with continued symptoms during treatment. The need for additional interventions is also supported by findings that therapist-guided internet treatments have better outcomes [6,7] and more patient adherence [6] than unguided interventions. Feelings of belonging and empowerment gained from the treatment might be key to patients’ completion of iCBT. Peer networks might be effective in increasing adherence to treatment.

The mental health sector has recently seen an increase in the use of peer support workers (PSWs) [17,18]. A peer support worker is a person with lived experience of mental health problems and rehabilitation who is employed in the mental health sector and becomes professionally active in recovery and support services for patients with mental health problems [19-21]. The PSW publicly identifies as a person who has received or is receiving mental health services [20] and has recovered enough from their mental illness to manage that illness and live a fulfilling life [19]. Peer support interventions focus on strengthening patients’ resources, rather than focusing simply on symptom reduction [22]. As peer supporters share their own experiences of the path from mental illness to recovery, they can function as role models and give patients hope [20,22]. This sharing of experiential knowledge might empower patients to become active agents in their own self-care [20,23]. Peer support services might also be effective in providing social support, which prevents isolation and acts as a buffer against stressors [20,24]. Through the helper-therapy principle, it has also been proposed that helping others might benefit PSWs themselves by strengthening their self-esteem, sense of empowerment, and confidence in their overall capability and ability to manage their illness [20].

The effectiveness of peer support services is promising; however, the evidence base is insufficient, and the results are mixed [19,25]. A recent systematic review [26] showed that the inclusion of peer support interventions in general clinical care is as effective as usual care conditions on traditional clinical outcomes, such as symptom severity and hospitalization rates, and that peer interventions have a positive effect on measures of hope, empowerment, and quality of life. Mahlke et al [22] found that the addition of peer support to standard treatment for patients with severe mental illness was related to higher scores of self-efficacy than standard treatment alone.

Recently, peer support services have been tested and offered in digital settings [21]. Some studies have investigated digital peer support in online interventions aimed at patients with bipolar disorders [27,28]. The participants in the supported groups were guided in the program by people with personal experiences of bipolar disorder. In both studies, adherence to treatment was significantly higher among patients receiving peer support [27,28]. A recent systematic review found preliminary evidence for the effectiveness of digital peer support on patients’ functioning, symptom reduction, and program utilization [21].

However, to date, few researchers have evaluated digital peer support in iCBT interventions. The results of a study in elderly adults with depression indicated that peer-supported CBT-informed intervention programs are acceptable and equivalent to individually delivered internet interventions. Less time from the therapist was needed in the group with peer support [29].

### Objectives

To our knowledge, no study has investigated peer support in primary care iCBT for adults with anxiety disorders. Therefore, the aim of this explorative study was to assess patients’ experiences, the feasibility, safety, and acceptability, and preliminary effectiveness of an 8-week primary care, peer-supported iCBT program for patients with anxiety disorders. The hypotheses were that contact with a peer supporter would enhance participants’ feelings of empowerment and increase their adherence to treatment.

## Methods

### Design

This was a single-arm, pre-post mixed methods intervention study to test the feasibility of an 8-week iCBT program for patients with anxiety disorders treated in primary care. This mixed methods study had a convergent design [30]. The intent of a convergent mixed methods design is to collect quantitative and qualitative data to allow the different methods to complement each other in terms of strengths and weaknesses and provide a fuller understanding of the research problem [30]. One motivation for using a mixed methods design in this study was to triangulate the research question using different methods to investigate whether the results of the different methods aligned with each other. The other motivation was to augment the quantitative data with qualitative data of participants’ experiences, as few prior studies have investigated using peer support in iCBT treatment, and thus to reveal new information that could be useful in future work. Outcome assessments were conducted at baseline, postintervention, and at the 3-month follow-up. Semistructured interviews were conducted postintervention to investigate the participants’ experiences of treatment. Quantitative and qualitative data were analyzed separately and integrated and interpreted during the discussion of the results. Multimedia Appendix 1 shows the flowchart of the study.

### Participants

The study was approved by the Regional Ethics Committee of Gothenburg (Dnr: 845-18). Participants were recruited from patients referred to a central unit for primary care iCBT in the Västra Götaland region, Sweden. Participants were adults aged 18 years or older with an anxiety disorder diagnosed according to ICD-10 [31]. The inclusion criteria were having reached the age of 18 years, having access to a computer with an internet connection, being able to speak and understand Swedish, and meeting the diagnostic criteria for an anxiety disorder (social anxiety, generalized anxiety disorder [GAD], panic disorder, obsessive compulsive disorder, or unspecified anxiety disorder). The exclusion criteria were having started pharmacological treatment for mental health problems or made major changes in the medication in the past few months, having serious suicidal ideation or suicide plans, having severe or complex comorbidity, or needing other care or receiving ongoing psychological treatment during the treatment period.

### Procedure

All participants were interviewed before and at the end of treatment by a psychologist using the structured diagnostic interview instrument, PRIME-MD [32]. Before starting treatment, all participants had a physical visit to the health care center and a somatic examination by a general practitioner (GP). The assessment interview was conducted by telephone upon referral from the health care center. The psychologist conducted an in-depth interview with the PRIME-MD and conducted a clinical assessment. After the interview, all patients who met the inclusion criteria were asked to participate in the study. They were asked verbally, and written information was sent by post to participants who wished to participate. The recruitment period for the study was 4 weeks in the spring of 2019. Of a total of 41 patients booked for an assessment interview, 21 met the inclusion criteria and were asked to participate. Of these 21 respondents, 15 agreed to participate. One of the initial 15 participants chose to withdraw from treatment before it began, and one chose to discontinue participation in the study after reading the information letter, but continued the usual iCBT. Two participants were excluded from data analysis because they were never active in the treatment program despite several reminders. Two participants failed to send in their written consent to participate in the study and were thus excluded from the data analysis. The results are thus based on information from 9 participants who agreed to the study and took part in the iCBT with additional peer support. Table 1 shows demographic variables of the participants.

**Table 1:** Demographic variables of patients (n=9) participating in the study.

Characteristics		Frequency, n (%)
**Age (years)**		
	18-25	4 (44)
	25-35	4 (44)
	35-45	1 (12)
	>45	N/A^a^
**Gender**		
	Male	4 (44)
	Female	5 (56)
**Employment status**		
	Employed	6 (67)
	Student	1 (11)
	Sick leave	2 (22)
**Previously received psychological treatment (lifetime)**		
	Yes	5 (56)
	No	4 (44)
**Other psychological treatment during time for iCBT^b^**		
	Yes (counseling)	1 (11)
	No	8 (89)
**Current medication for mental health problems during time for iCBT**		
	Yes (2 begun just before treatment)	3 (33)
	No	6 (67)

^a^N/A: not applicable.

^b^iCBT: internet-delivered cognitive behavioral therapy.

Quantitative data were collected through assessment forms sent to participants through a digital link sent to their email. The participants filled in assessments of the primary outcomes at the start of the treatment, at the middle of treatment, at the end of treatment, and 3 months after completing the treatment. The participants also completed a weekly assessment of symptoms of general psychological distress in connection with each new module in the treatment program. In addition, the participants regularly responded to a question regarding suicidal ideation as part of the weekly assessment of psychological distress. At the end of treatment, a follow-up assessment was made by the therapist in charge of the treatment to determine whether the participant still fulfilled the diagnosis received at the assessment interview before starting treatment. After completion of the treatment, qualitative data were collected. A trained research assistant, with no relationship to the participants, interviewed the participants about their experiences and attitudes toward treatment. The interviews were conducted by telephone 2 to 5 weeks after completion of treatment through a semistructured interview guided with open-ended questions such as “How did you experience the contact with a peer supporter?” The participants’ descriptions led the interviewer to add follow-up and in-depth questions such as “Can you tell me more?” The interviewer assured data reliability by repeatedly asking participants if they understood and by summing up what the participant had said. The interviews lasted 30 to 45 min. All interviews were audio-recorded and transcribed verbatim. The research assistant transcribed the interviews. Of the 9 participants who completed the treatment, only one declined to participate in the interview, and one responded to the interview questions in writing. The qualitative result is thus based on a total of 8 participants. In addition to quantitative measures and participant interviews, text messages sent from the peer supporters to the participants were collected and analyzed.

### Intervention

Two peer supporters were recruited through a Swedish patient organization, the National Cooperation for Mental Health in Gothenburg (NSPHiG). This organization has an established program for Swedish peer support education and a national platform of guidelines and frameworks. The organization has experience implementing peer support in psychiatric care and participated in recruiting and educating PSWs in this study. The 2 PSWs recruited for the study had experience working at an inpatient psychiatric clinic and were temporarily employed in primary care during the study. They worked 16 hours per week and supported 5 to 7 participants each. They had weekly scheduled meetings with a supervising psychologist, who is also the first author of this study. Supervision included discussing the treatment content, the participants’ answers on the questionnaires, and reflections on the written messages from the participants.

Peer supporters and participants interacted in the treatment program via asynchronous secure messages. Peer supporters were able to provide support and feedback on exercises in the iCBT program. The participants received a follow-up telephone call from the peer supporter in the middle of the treatment. In addition, the interactions in the text messages between peer supporters and participants were tracked by the supervising psychologist, who had joint access with the peer supporters to the treatment program in the digital system. The purpose of this was to ensure the safety of treatment. The psychologist, however, to stay true to how peer support operates in nondigital interventions [33] and to ensure that the content solely reflected the peer support intervention, did not try to influence the content of the peer supporters’ messages.

Participants also had limited contact with 2 licensed psychologists in the iCBT. Both psychologists worked at the participating clinic and were experienced in working with iCBT. All participants received a follow-up call from the psychologist after the completion of treatment. The psychologist also made telephone calls on the request of participants or if they thought it necessary (eg, if the psychologist noticed high scores on suicidal ideations or the participant remained inactive in the program for longer than 2 weeks—a routine intervention at the participating clinic).

In all cases, peer supporters had the most contact with the participants. The contact between the participants and the psychologists was limited to messages informing the participants that new modules had been activated in the treatment program. More detailed feedback on exercises and written messages was given only upon request from the participants. The peer supporters stated that they spent between 5 and 6 hours per week on the intervention. These hours included supporting the participants through the program as well as getting familiar with the treatment program and the content of each module. The psychologists spent roughly 10 to 15 min per participant per week (ie, 120 min per patient during the total treatment period).

The iCBT program used in this study was developed by Livanda-Internetkliniken AB for people affected by anxiety problems such as panic disorder, social phobia, and generalized anxiety. The program is based on both CBT methods and acceptance and commitment therapy (ACT) interventions and is a transdiagnostic program aimed at treating people with mild-to-moderate anxiety problems [34]. The program is designed as a course that includes education about symptoms common to anxiety disorders and as a training on different tools that have been shown to have a positive effect on such disorders. The program includes 13 different tools, and the treatment consists of 8 modules meant to be completed within 8 weeks. The tools presented are based on ACT principles such as exposure, acceptance, valued action, mindfulness, and defusion. The program contains psychoeducational text sections and video clips, assessments, and home assignments.

### Measurements

Anxiety was measured using the GAD 7-item scale (GAD-7) instrument. The instrument has 7 items measured on thresholds for mild, medium, and severe anxiety [35]. Symptoms of psychological distress were measured using Clinical Outcomes in Routine Evaluation 10 (CORE-10) [36]. The instrument consists of 10 items and has clinical cutoff scores for general psychological distress. Symptoms of depression were measured on the 9-item Montgomery-Åsberg Depression Rating Scale–Self report (MADRS-S) [37] with cutoff points for symptom severity. Empowerment was measured on the Empowerment Scale [38], which consists of 28 items. Acceptability was measured by using 4 questions, “To what degree have you experienced the treatment as helpful?” “How meaningful did you perceive the contact with the peer supporter?” “Would you recommend iCBT to someone else?” and “Would you recommend iCBT with peer support to someone else?” All questions were scored on a scale of 1 to 5. Higher values indicate greater acceptability.

### Data Analysis

#### Quantitative Data Analysis

The quantitative data analysis for repeated measures was performed using the Friedman analysis of variance (ANOVA) [39,40], which is a nonparametric correspondence to a one-way ANOVA with repeated measures. Post hoc analyses were performed using the Wilcoxon signed rank test for related samples. A Bonferroni-adjusted significance level was calculated to minimize the risk of type 1 error since multiple post hoc comparisons were made. The analysis was performed based on the intention-to-treat approach. The last observation carried forward was used to deal with missing data.

#### Qualitative Data Analysis

The qualitative data analysis was performed according to a thematic analysis [41]. The method was chosen because it provides the researcher with a flexible framework for finding themes and patterns in data [41]. The method is not bound to any theoretical foundation and can thus be used to analyze data both deductively and inductively. As this was an explorative study about the participants’ attitudes and experiences of a new treatment intervention, an inductive bottom-up approach was used to capture experiences and opinions as unconditionally as possible. The analysis was performed using a realist approach with the aim of identifying the manifest content of the participants’ attitudes and experiences. The first author’s pre-existing understanding of the topic is that of a working clinical psychologist and scientist as well as the project leader of the study. This might have influenced the understanding of the content of the interviews, but it also provided a deeper understanding of the intervention as a whole, which might have made the interpretation of the participants’ experiences deeper and more realistic. To ensure that the interpretation of the material was as close to reality as possible, the third and last authors were also involved in the interpretation process described below. In addition, the last author also read the original interview material to ensure a fit between the generated themes and the content of the interviews.

The data analysis was guided by the 6 steps described by Braun and Clarke [41]. The interviews were first read several times by the first author to become familiarized with the entire data set and formulate ideas about the initial codes and themes. All materials related to the research question were then coded by the first author. The software program, NVivo 12, was used to facilitate the coding process. After the initial coding, the codes were manually gathered by the first author into a thematic map, and preliminary themes and subthemes were identified. The themes were then reviewed by the first and last authors and revised when necessary. The first thematic map consisted of several themes and subthemes that were later refined. Examples of themes in this phase were experiences of peer support, effects of treatment, the treatment medium, the treatment program, and external factors. After this stage, the first author reviewed the codes and corresponding themes and subthemes with the third author, who had not been involved in the study design, recruitment, treatment, or initial analysis nor to that point had been familiar with the content of the interviews. After discussion, the initial themes were condensed into 3 main themes with corresponding subthemes. The codes were rechecked and reorganized by the first author according to the new themes. This process was guided by the dual criteria for judging internally homogenous and externally heterogeneous categories of [41]. Finally, the first author and last author read the original transcriptions of the interview material and reviewed the codes and corresponding themes and subthemes to ensure a fit between the interview content and the formulated themes.

#### Peer Supporters’ Text Messages

The text messages sent from the peer supporters to the participants were also analyzed using the same procedure as described above for the interviews. The first step of the analysis was performed by the first, fourth, and last authors and was compiled into themes by the first author. The fit between themes and content was rechecked by the last author.

## Results

### Quantitative Results

Quantitative results are based on information from a total of 9 participants who went through the iCBT with additional support from peer support. Five participants stated that they spent 0-2 hours per week on the treatment and 4 participants spent 2-4 hours per week. No participant dropped out after starting treatment. Some participants did not complete all of the modules, but they continued to have written contact with their peer supporter through messages in the program and thus remained active in the treatment. Two participants completed only one module, and 67% (6/9) completed more than half of the modules. Table 2 shows the number of modules completed for all participants.

Participants showed levels of anxiety and depression above the thresholds before treatment. Table 3 shows descriptive statistics for the different measurement points for GAD-7, MADRS-S, CORE-10, and the Empowerment Scale.

**Table 2:** Number of modules completed (n=9).

Participants, n (%)	Modules completed, n
2 (22)	1
1 (11)	4
3 (33)	5
2 (22)	6
1 (11)	8

**Table 3:** Descriptive statistics for measurement points on GAD-7, MADRS-S, CORE-10, and the Empowerment Scale.

Measurements		Median	Mean (SD)	Variance	Minimum scores	Maximum scores
**GAD-7^a^**						
	Pre	15	12.9 (4.3)	18.6	5	18
	Mid	9	9.8 (4.8)	23.2	4	18
	Post	5	6.8 (4.6)	20.9	2	13
	3-month follow-up	5	6.4 (4.6)	20.8	1	13
**MADRS-S^b^**						
	Pre	27	23.0 (6.4)	41.0	11	29
	Middle	23	19.9 (7.7)	59.9	5	28
	Post	15	15.8 (9)	80.9	4	28
	3-month follow-up	12	14.3 (10.4)	108.5	1	30
**CORE-10^c^**						
	Pre	22	22.9(5.6)	31.6	16	31
	Post	15	15.3(5.7)	32.5	8	25
**Empowerment Scale**						
	Pre	71	73.2 (9.7)	94.4	59	91
	Middle	72	73.8 (11.8)	138.7	59	95
	Post	80	77.6 (9.3)	86.0	62	90
	3-month follow-up	85	83.2 (12)	143.7	62	100

^a^GAD-7: generalized anxiety disorder 7-item scale.

^b^MADRS-S: Montgomery-Åsberg Depression Rating Scale–Self report.

^c^CORE-10: Clinical Outcomes in Routine Evaluation 10.

#### Anxiety

The results from Friedman test for GAD-7 showed a statistically significant difference between measurement points, *X^2^*_2_=11.6; *P*=.003. A post hoc analysis with a Wilcoxon signed rank test for related samples was performed with a Bonferroni correction applied, resulting in a significance level set at *P*<.017. Results from the post hoc analysis showed a statistically significant reduction in anxiety symptoms from pretest to 3-month follow-up (*Z*=−2.552; *P*=.01; *r*=0.60) as well as from pretest to posttest (*Z*=−2.668; *P*=.01; *r*=0.63). There was no statistical difference in anxiety symptoms between postmeasurement and 3-month follow-up (*Z*=−0.170; *P*=.87; *r*=0.04).

#### Depression

For MADRS-S, the results from Friedman test showed a statistically significant difference between measurement points (*X*^2^_2_=9.9; *P*=.01). Results from the post hoc analysis with the Wilcoxon signed rank test, with the Bonferroni correction applied (*P*<.017), showed a statistically significant reduction in depressive symptoms from pretest to the 3-month follow-up measurement (*Z*=−2.429; *P*=.02; *r*=0.57) and from pretest to posttest (*Z*=−2.521; *P*=.01; *r*=0.59). There was no statistical difference in depressive symptoms between postmeasurement and 3-month follow-up for MADRS-S (*Z*=−0.491; *P*=.62; *r*=0.12).

#### Psychological Distress

Results from the Wilcoxon signed rank test for CORE-10 showed a statistically significant reduction in symptoms of psychological distress from pretest to posttest (*Z*=−2.524; *N-*ties=8; *P*=.01; *r*=0.59). The 3-month follow-up test for CORE-10 was not assessed since the participants filled in this measurement in connection with new modules of the treatment program and, by the 3-month follow-up, no longer had access to the treatment program.

#### Empowerment

The results from Friedman test for the Empowerment Scale showed a statistically significant difference between measurement points (*X*^2^_2_=10.1; *P*=.01). Results from the post hoc analysis with a Wilcoxon signed rank test with the Bonferroni correction (*P*<.017) showed a statistically significant increase in experienced empowerment from pretest to 3-month follow-up measurement (*Z*=−2.547; *P*=.01; *r*=0.60). There was no statistically significant difference between pre- and postmeasurement with the Bonferroni correction applied (*Z*=−2.082; *P*=.04; *r*=0.49) or between postmeasurement and 3-month follow-up with the Bonferroni correction applied (*Z*=−2.371; *P*=.02; *r*=0.56).

### Clinically Significant Improvement

Clinically significant improvement was determined by comparing the scores on GAD-7 and MADRS-S against the thresholds on the respective scales for mild, moderate, and severe anxiety and depression at the start of treatment and at the end of treatment. Of the 9 patients, 8 were considered “improved” on the severity of their symptoms of anxiety; 4 were considered “improved” on symptoms of depression based on the thresholds for mild, moderate, and severe depression on MADRS-S; 5 were considered “unchanged” for symptoms of depression, although 1 did not meet the cutoff threshold for depression at startup or at the 3-month follow-up. Table 4 shows the changes in the cutoff scores for the GAD-7 and MADRS-S.

**Table 4:** Clinically significant improvement.

Participant	Symptom severity for anxiety based on cutoff scores on GAD-7^a^	Improvement GAD-7	Symptom severity for depression based on cutoff scores on MADRS-S^b^	Improvement MADRS-S
1	From severe to moderate	Improved	From moderate to moderate	Unchanged
2	From mild to absent	Improved	From absent to absent	Unchanged
3	From moderate to mild	Improved	From moderate to mild	Improved
4	From severe to absent	Improved	From mild to absent	Improved
5	From moderate to mild	Improved	From mild to mild	Unchanged
6	From severe to mild	Improved	From mild to absent	Improved
7	From severe to moderate	Improved	From moderate to moderate	Unchanged
8	From mild to moderate	Unchanged (worsening)	From moderate to moderate	Unchanged
9	From severe to mild	Improved	From moderate to absent	Improved

^a^GAD-7: generalized anxiety disorder 7-item scale.

^b^MADRS-S: Montgomery-Åsberg Depression Rating Scale–Self report.

Based on the diagnostic interview with Prime-MD conducted at the start and the end of the treatment, 5 participants no longer met the criteria for their main anxiety diagnosis after treatment; 4 were assessed as still meeting the criteria for their main anxiety diagnosis, one of whom was referred back to the health care center for further treatment. With that one exception, none of the participants were considered in need of further treatment for anxiety. Table 5 shows the participants’ diagnoses at the start and end of the treatment.

At the 3-month follow-up assessment, 4 participants stated that they had sought continued care for their mental health; 3 stated that they had started some form of counseling, and 1 had started pharmacological treatment for mental illness. Of the 4 participants who sought continued care after termination of iCBT, 1 was classified as unchanged, while 3 were classified as improved according to the results above.

**Table 5:** Diagnosis at start and end of treatment assessed by the diagnostic interview, PRIME-MD.

Participant	Diagnosis at start of the treatment	Meets criteria for diagnosis after treatment	Referred for further treatment
1	Anxiety, unspecified Depressive episode, unspecified	Yes No	N/A^a^
2	Panic disorder Anxiety, unspecified Specific phobia (emetophobia)	No No Yes	N/A
3	Panic disorder Generalized anxiety disorder Social phobia Depressive episode, unspecified Obsessive compulsive disorder Eating disorder, unspecified	No Yes Yes Yes No Not assessed	N/A
4	Social phobia Generalized anxiety disorder	No Yes	N/A
5	Anxiety, unspecified Depressive episode, unspecified	No No	N/A
6	Generalized anxiety disorder Social phobia	Yes Yes	N/A
7	Generalized anxiety disorder Social phobia Depressive episode, mild	Yes Yes Yes	Referred to health care center
8	Generalized anxiety disorder Recurring depressive episode, moderate	Yes Yes	N/A
9	Anxiety, unspecified	No	N/A

^a^N/A: not applicable.

### Safety and Acceptability

No serious adverse events were reported during the treatment period or in the interviews with the participants. The participants (n=9) scored a median value of 4 (on a scale of 1-5) on the question “To what degree have you experienced the treatment as helpful?” The participants also scored a median value of 3 (on a scale of 1-5) on the question “How meaningful did you perceive the contact with the peer supporter?”

For the question “Would you recommend iCBT to someone else?” 7 of the 9 participants stated that they would recommend it, 1 would not, and 1 did not know or had no opinion. For the question “Would you recommend iCBT with peer support to someone else?” 7 of the 9 participants answered affirmative and 2 stated that they did not know or had no opinion.

### Qualitative Results

Three main themes with associated subthemes were generated from the qualitative results: real contact in an online world (subthemes: “Support and encouragement” and “Sharing experiences provides personal contact”); empowering experiences (subthemes: “Changes in psychological well-being,” “An ongoing task,” and “Acquired psychological strategies”); and being behind the wheel (subthemes: “Flexibility and responsibility” and “Barriers to treatment”).

The quotes in the text have been translated into English.

#### Real Contact in the Online World

This theme relates to the participants’ experiences of their contact with the peer supporter and their thoughts on what had been helpful or not about having the peer support in treatment.

##### Support and Encouragement

The participants generally described having had a good experience with the peer supporter. Several described their perception of peer support as very good and said that contact with a peer supporter could help someone to see another side to anxiety and to imagine the possibility of feeling better. Several participants said that it felt good when the peer supporter checked in on them and showed that they were there by emailing every week, asking them questions about how things had been going. They said it was useful to know there was someone there who they could turn to. Many thought it was nice to be able to write about anything they felt like and that the treatment program felt more real when there were real people to write to:

I absolutely believe that it is a very good idea and that you know that you are not alone and that it is possible, there is a second side to the problem as well. You can crawl out of this, so that’s what I think but it was, it was a good experience on the whole.

Participant #8

I thought it was very, it was nice that the person, the peer supporter noticed if I had written that—I haven't had anyone to talk to […] and then he wrote directly saying that you can write to me when it is needed, and then I wrote to him, so I thought it was very nice, to just be able to write to someone like that.

Participant #3

Several participants were able to point to situations where contact with peer support was extra helpful; however, some stated that they had not had much contact with their peer supporter. A couple of participants described wanting more verbal contact with the peer supporter and thought that this had facilitated their connection with each other. In line with this, some participants described how it had been extra helpful when they were able to speak with their peer supporter on the phone. One participant asked for a physical meeting with the peer supporter at the beginning of treatment and thought this had facilitated their connection. One other participant, however, felt that digital contact reduced the pressure of social settings and made it easier to open up. Another described having wanted to know more about the peer supporter's background and concrete experience. In line with this, another participant described how the contact was made more difficult because of uncertainty about the peer supporters. Yet another participant felt that the treatment worked equally well without peer support.

##### Sharing Experiences Provide Personal Contact

Many of the participants felt that it was positive to share experiences with someone who had been in a similar situation. Some described situations during treatment when it had been helpful when the peer supporter shared how they had handled similar situations in the past:

The peer supporter could write that I’ve also felt like that sometimes, and I usually think this way and this, that if you are afraid to say something that will sound wrong, then try and do it and see what happens.

Participant #3

Several of the participants said that it felt like a more personal contact to talk to someone with similar experiences than to health care professionals (therapists, psychologists, or doctors). The peer supporter was seen as a fellow human being and someone who could understand their problems differently than a therapist because of their personal experiences:

It was pretty nice to talk to someone. She had gone through everything, and it wasn’t like someone or a therapist was in charge, but maybe a fellow human being, who knows where you are at.

Participant #1

#### Empowering Experiences

This theme relates to the participants’ experiences of being strengthened in themselves and how the treatment contributed to their feeling able to handle their anxiety in a different way.

##### Changes in Psychological Well-Being

The majority of the participants described feeling less anxious in general after the treatment and said that situations that used to provoke anxiety did not do so anymore. Many participants also described changes in cognition as they thought of their emotions as “just feelings” and thus felt that they could handle their anxiety differently than before starting treatment:

Well that, in fact, it might not be so dangerous. It’s just a feeling. It can be very difficult, and to try to tell yourself that it’s actually just a feeling, you probably won’t not die anyway.

Participant #1

Several participants described how the treatment had helped them do things they had previously avoided because of their anxiety. They described pushing themselves to talk in situations that made them feel uncomfortable, daring to make mistakes, or to state their opinion more clearly. Some described how going through treatment had created a positive feedback loop and that doing things they had previously avoided made them feel stronger about themselves. Some also described how exposing themselves to situations they had previously avoided helped them to realize that those things were not as dangerous as they had thought and to realize that if they did not try, things would not get better:

…when I had anxiety, I often put things off. I mean I didn’t want to meet people, but now I force myself to just meet people, because it is not getting better from me not doing it.

Participant #4

The treatment has helped me to be able to do things that I previously was anxious about. So it has strengthened me, it has strengthened other parts of my person and my inner well-being, which has made me less uncomfortable in those situations that made me uncomfortable in the past.

Participant #9

A couple of the participants felt that through the treatment they had realized that they were not the only ones to deal with anxiety, and this realization made them feel less alone. They also felt that they could think of their unhelpful thoughts more as symptoms of anxiety than reflections of real conditions, and could therefore feel less odd.

Several participants described feeling they had taken hold of themselves by deciding to go through treatment, and a couple described having clearer thoughts about themselves as the people who had to deal with their problems.

##### An Ongoing Task

Most participants described feeling that they would continue working on themselves even after treatment. Some pointed to different interventions in the treatment program that they were going to remind themselves to continue using. Others stated that they would continue to do exercises from the program to become better at taking care of themselves:

[There were] some practical exercises to do, and to bring those with you, to keep doing those exercises when needed. Or maybe not even when needed, but on a regular basis.

Participant #9

##### Acquired Psychological Strategies

Many participants described the program as having given them helpful tools and new knowledge about thoughts, feelings, and physical reactions to anxiety. Some participants emphasized having a different perspective on their own thoughts and having learned to question them. Other strategies that patients acquired were being able to put their thoughts and feelings into words and to set goals for themselves. The participants mentioned helpful interventions such as breathing exercises or postponing rumination to a set time.

#### Being Behind the Wheel

This theme concerns the participants’ perceptions of the form of the treatment and the perceived advantages and disadvantages of mediating the treatment digitally.

##### Flexibility and Responsibility

Many participants had a positive attitude toward the treatment program, and several thought the treatment had helped them. None of the participants felt that the treatment had resulted in a negative change for them. However, a few participants felt that the treatment was not suitable for them.

Several participants emphasized the increased flexibility of the digital treatment and appreciated being able to access the treatment when it suited them and to reflect on the content at their own pace:

It was actually the whole concept of having someone all the time that you can have contact with and at the same time be able to read up on and do things yourself, but at the same time be able to write to someone if you feel that you need it.

Participant #3

In contrast, a few participants described how the flexibility of the treatment could also be a disadvantage, as they postponed working with the treatment, had less time to sit with the program than they had intended, or felt too tired to engage with the program after a day of working. In line with this, some participants reasoned that the digital medium for treatment placed greater demands on their own responsibility and self-awareness. They reasoned that this might place a greater demand on patients to have a functional everyday life and that the treatment might be more suitable for patients with less severe mental illness:

My first CBT treatment in group, for example, did not work because I was too bad and what was required there was way beyond what I could handle, so I would say that maybe it is something to try when things start to stabilize so that you are able to take that responsibility yourself.

Participant #8

##### Barriers to Treatment

The participants also described some difficulties related to the digital form of treatment. Some participants felt that there was too much information in the treatment program to read and listen to. Several participants also mentioned that the pace of the treatment was too quick, and they wished for more time to go through the program. A few participants described having difficulty engaging in the treatment because it came at an inappropriate time in life, they were not prepared for how much the treatment would require of them, and they found it hard to take charge of doing things for themselves. Some participants said that they had wished for more verbal contact with the therapist or the peer supporter and thought this might have facilitated their engagement with the treatment program:

It was nothing that suited me because a lot of what I needed to do was a little more, I got stuck in trying to understand it myself and doing it, and it was that, that was the problem from the beginning, so it’s like, I never got started so to speak.

Participant #7

### Peer Supporters’ Text Messages

Interviews with peer supporters showed that they were satisfied with their role in the treatment program. The peer supporters generally felt that the pace of their support could have been quicker and thought they could have supported 8 to 12 patients per day rather than 5 to 7.

The qualitative analysis of the peer supporters’ text messages to the participants (n=81) resulted in 3 themes: reinforcement of resources, being present for the patient, and being personal.

Significant for the theme “Reinforcement of resources” was how the peer supporters reinforced the participants’ work with the treatment by paying attention to and encouraging the participants to work with the treatment program. They reinforced participants’ engagement in positive behaviors or behaviors related to their valued direction or goals (such as self-exposure to anxiety or using strategies from the treatment program) and encouraged them to express and share their opinions about the treatment’s form, content, and specific exercises. The peer supporters also invited dialogue with participants through questions such as “What do you think about the treatment program?” or “How do you feel you are doing with the treatment?” The theme “Being present for the patient” represents the various ways in which peer supporters showed the participants that they were to support them. The peer supporters validated the participants’ difficulties, expressed empathy, and encouraged them to get in touch with them if they had any questions or difficulties. The peer supporters asked about the participants’ lives, but also created connection with the participants by using everyday expressions such as “Happy weekend” or “I wish you a happy Easter.” The peer supporters also encouraged the participants to continue working with the treatment program through written reminders and by prompting upcoming exercises. The theme “Being personal” refers not only to how the peer supporters shared their own thoughts and opinions about the treatment program and various exercises that they thought were helpful for them, but also to how they shared their own experiences of dealing with and handling similar difficulties in various situations. The peer supporters also made use of self-disclosure by telling the participants about situations they found difficult and emphasizing self-acceptance and self-compassion when confronting difficulties in life.

## Discussion

### Principal Findings

To our knowledge, this is the first study to embed peer supporters in primary care iCBT treatment for anxiety. We used a naturalistic single-arm mixed methods approach to investigate the feasibility, safety, experiences, acceptability, and preliminary effectiveness of a peer-supported 8-week iCBT program on anxiety, depression, psychological distress, empowerment, and adherence to treatment. The results show that such a program is feasible and that participants appreciated the peer supporters’ help. The study was conducted in a clinical setting and thus supports the feasibility of adding peer support to iCBT treatment in real-world settings. A common criticism of efficacy studies (ie, studies conducted in research settings with rigorous design methods and with strict selection of research participants) is that research conditions do not accurately simulate the real world and thus have high internal validity but limited external validity [42]. Our sample was largely representative of the patient profile in primary care, which includes a range of socioeconomic and clinical backgrounds. This study also provides evidence of the general feasibility and effectiveness of iCBT in clinical (specifically primary care) settings, as only a few studies on iCBT have been conducted in clinical settings [12]. The quantitative results further support previous research showing that iCBT is effective both for mild-to-moderate problems [5-8] and for more severe mental illness [43]. The participants in this study had symptoms of anxiety and depression above the cutoff thresholds, indicating that iCBT treatment might be effective for more severe psychological distress.

The quantitative and qualitative results of this study support the presumptive positive effect of peer support. The quantitative results showed a significant reduction in anxiety and depression after treatment, which was maintained at the 3-month follow-up assessment. There was also a significant reduction in general psychological distress at the end of the treatment. Statistical data were also supported by clinical measures and qualitative data. The participants felt that the treatment contributed to their feeling of being able to handle their anxiety differently. Participants also declared that they felt their lives had expanded and, that after treatment, they could do things they had previously avoided.

The combined results show an increase in the participants’ sense of empowerment connected both to their contact with the peer supporter and the actual tools in the iCBT program. Several participants described how contact with a peer supporter helped them see that it was possible to feel better. The treatment program was seen as helpful because it provided tools to overcome psychological barriers associated with anxiety, and overcoming these barriers allowed the participants to feel more empowered. The quantitative results also showed a significant increase in the participants’ sense of empowerment between the start of treatment and the 3-month follow-up after completion. The quantitative and qualitative results in this study showing increased empowerment are in line with previous research showing that peer support interventions are related to increased measures of self-efficacy, hope, and empowerment [22,26]. These observations reflect several key learning points in peer-supported interventions, such as focusing on strengthening the patient’s resources. In addition, when peer supporters share experiences of their own paths from mental illness to recovery, they can function as role models and thus provide hope for patients.

Prior research shows that guided iCBT is generally more effective than unguided [6,7]; however, little is known about what contributes to effective guidance in internet-mediated treatment. The internet provides enormous possibilities for disseminating evidence-based psychological treatment; however, it provides little personalized contact and might seem more effortful to patients. This possible disadvantage was evident in this study with participants saying that although internet-mediated treatment provides flexibility, it demands more responsibility from patients than face-to-face treatment. This is the only study that we know of that has qualitatively analyzed participants’ experiences of peer support in iCBT, but several prior qualitative studies have analyzed participants’ experiences of therapist-supported iCBT. The results of this study regarding patient perceptions of the treatment medium are in line with previous qualitative studies. The flexibility of iCBT is often perceived both positively, by contributing to patients’ experiences as the primary agents of their own change and their ability to choose when and how to receive the treatment [44-46], and negatively, by placing more responsibility on patients, which can be experienced as demanding and might contribute to difficulties in engaging with the treatment [47,48]. Many studies also emphasize support from the therapist as an important factor in treatment outcomes [44-46,49].

Based on this study’s results, one factor that can contribute to effective guidance in psychological internet treatment might be more personal and self-disclosing messages, which become even more important in a digital context than in normal face-to-face treatment, where alliance-forming factors such as body language, tone, and implicit validation strategies are lost. The analysis of the text messages sent from peer supporters to participants in the treatment program in this study shows how peer supporters made use of self-disclosure, shared their own experiences of dealing with difficulties in life, and shared their personal reflections on the content and tools they thought were helpful in the program. The usefulness of this was strengthened by the qualitative results, in which several participants emphasized the personal connection they felt when they were able to share their difficulties with someone who had similar experiences.

Adherence to treatment in this study was above that commonly seen in other studies on iCBT, with a recent systematic review and meta-analysis showing an average dropout rate of 15.7% in guided iCBT treatment for psychiatric and somatic conditions [7]. In this study, despite its small sample size, no participant dropped out of treatment and 67% (6/9) completed more than half of the treatment modules. These results are in line with previous studies on digital peer specialists in internet treatment [21,27,28], supporting the hypothesis that peer support can enhance treatment engagement. Moreover, in this study, less time was required by the therapist than usual in iCBT at the participating unit since the study was designed to limit support from the clinicians in favor of peer support. These results support the feasibility of this study. This is also in line with a previous study on peer-supported iCBT interventions, which showed that less support from therapists was needed in the peer-supported group [29]. A recent meta-analysis on iCBT for anxiety and depression disorder reported that therapists reporting spending from 18 to 352 min per patient ranged over the treatment period, indicating large general between-therapist differences in time allotted to iCBT [50]. In this study, clinicians spent roughly 120 min per patient over the entire treatment period. Less time from the therapist can potentially increase the scalability of iCBT and increase access to care for patients.

The qualitative results from this study also pointed to some improvements that could be made in the design of the study. Some participants stated that their contact with the peer supporter was not fully utilized. Stated reasons for difficulties in engaging with the peer supporter included a lack of knowledge about the peer supporter’s background and training, and a treatment period too short to create a real connection with each other. Some participants and peer supporters (in discussions during supervision) suggested that more verbal contact could be useful for creating a sense of connection, especially early in treatment. Prior research suggests that to be effective, it is important that peer support services reflect cultural diversity [20]. Research on self-help groups shows that when participants perceive the other people in the group to be similar to themselves, they are more likely to continue attending the group [20]. For administrative reasons, this study had only 2 PSWs, which limited their cultural diversity and might have influenced the perceived helpfulness of the peer supporters for some participants.

Future studies could contribute further by exploring how interactions between participants and peer supporters can be enhanced.

### Limitations

This study was an uncontrolled feasibility study, so it is not possible to rule out factors other than the treatment affecting the outcomes. We also do not know whether similar results would have been seen in iCBT treatment without peer support for this specific group of participants. The study also has a limited sample size, which limits both the conclusions that can be drawn from the quantitative assessments and the transferability of the qualitative data. Data saturation is defined as the point when additional data no longer add new information to the analysis, and it is used to determine when the recruitment of new interview participants can be terminated. The themes formulated in this study thus cannot claim transferability but can guide the development of future peer-supported services embedded within iCBT treatments. Despite the limited sample size in this study, a clear strength is the combination of several different analytic methods, including statistical analysis, clinical measures, and qualitative analysis, all of which support the conclusion that peer support is a feasible addition to iCBT.

In light of its limitations, this study should be replicated in larger sample sizes, different populations, different contexts, and randomized controlled trials.

### Conclusions

This study supports the feasibility of adding peer support to iCBT for adults with anxiety disorders. The results suggest preliminary support for the effectiveness of peer support on patient empowerment, reduction in anxiety, depression, and psychological distress, and adherence to treatment. Qualitative results also suggest that clinicians may be more effective by allowing themselves, such as PSWs, to be more personal and self-disclosing in their messages in the treatment program. Peer support might therefore contribute to more effective guidance in internet-based psychological treatment and might counter the loss of alliance-forming factors such as body language, tone, and implicit validation present in physical encounters. Future studies should validate the findings of this study with larger sample sizes and randomized controlled trials.

## Appendix

Multimedia Appendix 1 Flowchart of the study.

## Abbreviations

ACT: acceptance and commitment therapy
ANOVA: analysis of variance
CBT: cognitive behavioral therapy
CORE-10: Clinical Outcomes in Routine Evaluation 10
GAD: generalized anxiety disorder
iCBT: internet-delivered cognitive behavioral therapy
MADRS-S: Montgomery-Åsberg Depression Rating Scale–Self report
NSPHiG: National Cooperation for Mental Health in Gothenburg
PSW: peer support worker
