# Supplementary material for: Primary Care Peer-Supported Internet-Mediated Psychological Treatment for Adults With Anxiety Disorders: Mixed Methods Study
Source: JMIR Form Res. 2020 Aug 20;4(8):e19226. doi: 10.2196/19226 (PMC7471888; doi:10.2196/19226)
Supplement: Multimedia Appendix 1 [file formative_v4i8e19226_app1.docx]

Single-arm mixed methods pre-post intervention study

Quantitative Baseline Assessment (n=9)

Quantitative 3-month Follow-up Assessment

Quantitative Post-intervention Assessment (n=9)

8 week intervention period (iCBT treatment with peer support)

Integration of quantitative and qualitative data 🡪 interpretation

-Enhance interpretation of the quantitative outcome results

-Understand how participants experience the treatment

Separate Qualitative Analysis (Thematic analysis)

Separate Quantitative Analysis (Friedmans ANOVA)

Qualitative data collection by semi-structured interviews (n=8)
